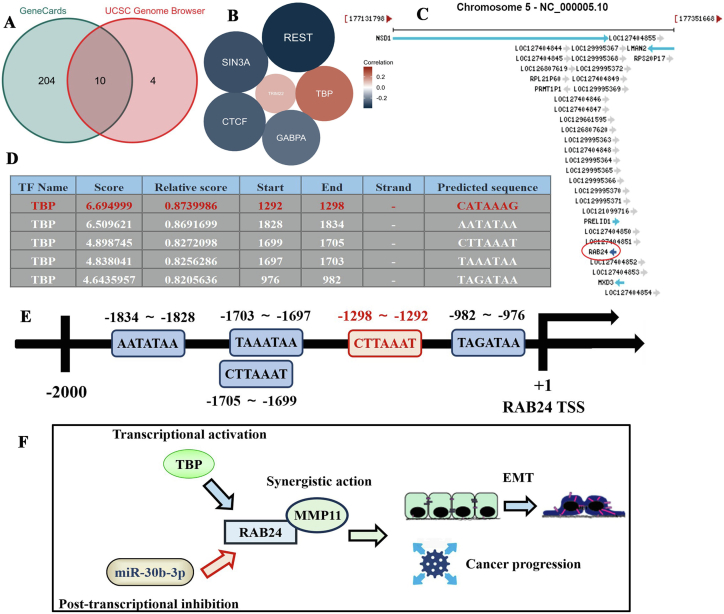# RAB24 maps comprehensive clinical landscapes and mediates tumor malignant progression under the epigenetic regulation of miR-30b-3p and MMP11 in clear cell renal cell carcinoma

**DOI:** 10.1016/j.gendis.2025.101869

**Published:** 2025-09-24

**Authors:** Fangshi Xu, Qiao Du, Gang Li, Lingyu Guo, Jiancang Ma, Zhenlong Wang

**Affiliations:** a Department of Vascular Surgery, The Second Affiliated Hospital of Xi'an Jiaotong University, Xi'an, Shaanxi 710004, China; b Department of Urology, The Second Affiliated Hospital of Xi'an Jiaotong University, Xi'an, Shaanxi 710004, China; c Department of Urology, Baoji People's Hospital, Baoji, Shaanxi 721000, China; d Department of Medicine, Xi'an Jiaotong University, Xi'an, Shaanxi 710004, China

Renal cell carcinoma (RCC) is the third most common urological malignancy. Due to its high metastatic potential, approximately 20% of patients present with metastases at diagnosis. Autophagy, a form of programmed cell death (PCD) characterized by autophagosome formation and lysosome-mediated degradation, plays a crucial role in tumor biology. Given that membrane dynamics are essential throughout the autophagic process, RAB proteins, which are key regulators of membrane trafficking, have garnered increasing attention. Among them, RAB24 stands out as an atypical member due to its roles in basal autophagy and endosomal degradation. However, its function in clear cell RCC (ccRCC) remains largely unexplored, prompting us to investigate this research gap.

The detailed materials and methods are provided in Supplementary file 1. Prior to multi-omics analysis, we compiled a comprehensive autophagy-related (AR) gene set (*n* = 612) using the HADb and MSigDB databases (Fig. 1A). Differential expression analysis identified 95 AR genes (Fig. 1B), from which four candidates were selected using LASSO regression and SVM-RFE algorithms (Fig. 1C). Notably, only RAB24 showed a strong correlation with the epithelial–mesenchymal transition (EMT) process (Cor = 0.323) (Fig. 1D).

**Figure 1:**
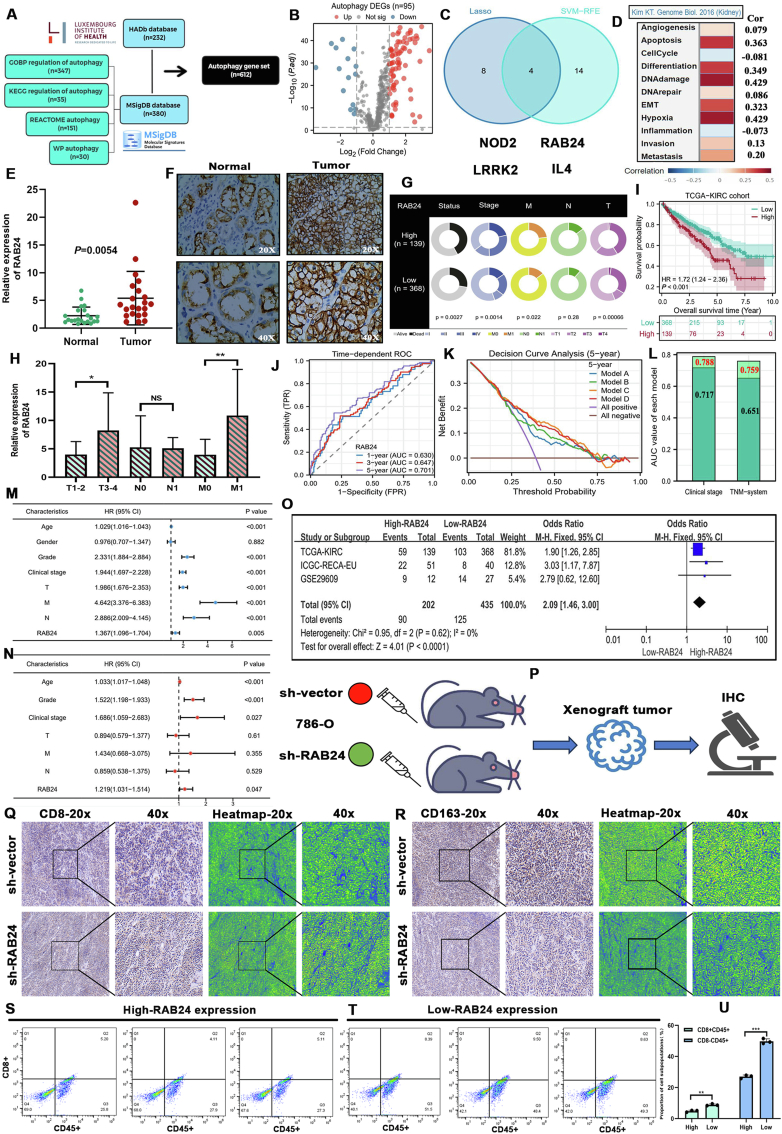
RAB24 is indicative of the prognostic and immune status of ccRCC. **(A)** Construction of a comprehensive AR gene set. **(B)** Screening autophagy DEGs between normal and ccRCC samples (the absolute value of Log2FC is greater than 1.5). **(C)** The intersection part between two machine learning algorithms. **(D)** The quantified relationships between multiple biological processes of RCC and RAB24 expressions based on the CancerSEA database. **(E)** The differences in mRNA expressions of RAB24 between normal and ccRCC samples (XJTU cohort). **(F)** The histological expressions of RAB24 between normal and ccRCC samples (XJTU cohort). **(G)** The relationships between RAB24 expression and the clinicopathological characteristics of ccRCC (TCGA-KIRC cohort). **(H)** The expressive differences of RAB24 between early and late clinical stages (XJTU cohort). **(I)** The overall survival difference between the high- and low-RAB24 expression groups. **(J)** Time-dependent accuracy of RAB24 for predicting OSR. **(K)** The decision capacities of the four prognostic models based on DCA analyses. Model A (blue line) represents the prognostic model based on clinical stage. Model B (green line) represents the prognostic model based on the TNM system. Model C (orange line) represents the prognostic model combined with clinical stage and RAB24 expression. Model D (red line) represents the prognostic model combined with the TNM system and RAB24 expression. **(L)** The improvement in the predictive accuracy of traditional prognostic models after introducing RAB24 expression. **(M, N)** The identification of RCC-independent prognostic factors through Cox univariate (blue) and multivariate (red) analyses. **(O)** The prognostic meta-analysis of three cohorts. **(P)** The detection of immunological markers on mouse xenograft tumors. **(Q, R)** The immunohistochemical staining of CD8 and CD163 in mice xenograft tumors. **(S)** The results of flow cytometry analyses on three tumor samples with high RAB2 expression. **(T)** The results of flow cytometry analyses on three tumor samples with low RAB2 expression. **(U)** The differences in the infiltration levels of CD8^+^ CD45^+^ cells and CD8^–^ CD45^+^ cells between tumor samples with high- and low-RAB24 expressions. These experiments were repeated independently three times. Cell quantitative analysis was conducted using a high magnification microscope (100-fold) of five random visual fields. Differences between different groups were assessed using Student's *t*-test. AR, autophagy-related; DEGs, differentially expressed genes; XJTU, Xi'an Jiaotong University; DCA, decision curve analysis; sh-RAB24, short hairpin RNA targeting RAB24; IHC, immunohistochemistry; CD45, a common antigen of mature leukocytes; ∗∗∗*p* < 0.001.

RAB24 was significantly up-regulated in tumor samples as determined via polymerase chain reaction (PCR) and immunohistochemistry (IHC) analyses (Fig. 1E and F). High RAB24 expression was associated with advanced clinical stage and increased incidence of metastasis (Fig. 1G and H), as well as poorer survival outcomes (Fig. 1I). Its predictive value for overall survival in ccRCC patients was moderate (AUC = 0.630–0.701; Fig. 1J). Importantly, RAB24 improved the net clinical benefit (Fig. 1K) and enhanced the predictive accuracy of existing prognostic models (Fig. 1L). Multivariate analysis identified RAB24 as an independent prognostic factor for ccRCC (Fig. 1M and N). These findings were further validated in the ICGC-RECA-EU and GSE29609 cohorts (Fig. S1A–S1H). A meta-analysis confirmed that high RAB24 expression significantly increased the risk of poor prognosis (Fig. 1O, OR = 1.46). Collectively, RAB24 represents an important supplement to the prognostic assessments of patients with ccRCC.

As for immune effects, patients with ccRCC with high RAB24 expression exhibited reduced CD8^+^ T cell infiltration and decreased cytolytic activity, suggesting that RAB24 overexpression may impair anti-tumor immune responses (Fig. S1I and S1J). Interestingly, no significant difference in overall immune scores was observed between the high- and low-RAB24 expression groups (Fig. S1K). To further investigate the immunological role of RAB24, we performed IHC analyses on xenograft tumors from mouse models (Fig. 1P). Tumors derived from the RAB24-knockdown group showed strong CD8 staining, whereas CD163 expression was weakly positive (Fig. 1Q and R). These results suggest that silencing RAB24 enhances CD8^+^ T cell infiltration while limiting macrophage accumulation. To validate these observations in human samples, flow cytometry was conducted on six ccRCC tumor specimens with varying RAB24 expression levels (Fig. 1S–U). In high-RAB24 tumors, CD8^+^ T cells accounted for only 4.11%–5.20% of immune cells (Fig. 1S), whereas low-RAB24 tumors exhibited higher CD8^+^ T cell proportions, around 8% (Fig. 1T). These findings support the notion that RAB24 overexpression contributes to an immunosuppressive tumor microenvironment.

Functionally, overexpression (OE-RAB24) and knockdown (sh-RAB24) of RAB24 in 786-O and Caki-1 cells were confirmed via Western blot (Fig. S2A). Colony formation and Transwell assays demonstrated that RAB24 overexpression promoted proliferation, migration, and invasion, while its knockdown suppressed these malignant behaviors (Fig. S2B–S2D). Additionally, RAB24 overexpression enhanced EMT-related processes (Fig. S2E). RAB24 knockdown significantly suppressed tumor growth in xenograft models *in vivo* (Fig. S2F–S2H). Collectively, these findings indicate that RAB24 functions as a pro-tumorigenic factor in ccRCC.

A previous study by our team demonstrated that miR-30b-5p promoted ccRCC tumorigenesis by targeting Rab3D.^1^ This finding led us to investigate whether miR-30b-3p, another mature miRNA derived from the same precursor, also contributes to ccRCC progression (Fig. S2I). Using the TargetScanHuman database, we predicted a binding site for miR-30b-3p in the 3′-UTR of RAB24, which was subsequently validated through a dual-luciferase reporter assay (Fig. S2J–S2K). Functional assays revealed that miR-30b-3p mimics significantly reduced the proliferation, migration, and invasion of ccRCC cells, while RAB24 overexpression partially rescued these inhibitory effects (Fig. S2L–S2N). Similarly, miR-30-3p inhibitors enhanced the proliferative, migrative and invasive abilities of 786-O and Caki-1 cells, while RAB24 deletion could partially offset the pro-carcinogenic effects of miR-30-3p inhibitors (Fig. S3A–S3C).

Further *in vivo* experiments were conducted to validate the regulatory relationship between miR-30b-3p and RAB24. Stable transfection of miR-30b-3p mimics into 786-O cells significantly inhibited tumor growth in a subcutaneous xenograft mouse model (Fig. S3D). Tumors from the miR-30b-3p group were significantly smaller in both weight and volume compared to those from the controls (Fig. S3E–S3F). Following tumor excision, immunohistochemical staining was performed to assess RAB24 and proliferating cell nuclear antigen (PCNA) expression, with the latter serving as a key marker of cellular proliferation. The results showed a significant reduction in RAB24 expression in tumors from the miR-30b-3p group (Fig. S3G), accompanied by a concordant decrease in PCNA levels (Fig. S3H). These findings establish a novel regulatory pathway in ccRCC, referred to as the miR-30b-3p/RAB24 axis.

Interestingly, the expression levels of classical autophagy markers, including p62, LC3, and Beclin1, remained unchanged following RAB24 modulation (Fig. S4A and S4B). Transmission electron microscopy (TEM) also confirmed that regardless of RAB24 overexpression or deletion, no obvious alteration in autophagy intensity was observed (Fig. S4C). This suggests that, despite RAB24's established role in autophagy, its autophagic function may be impaired or context-specific in tumor cells. Two possible explanations may account for this observation. First, RAB24 plays an essential role in mitotic regulation, including chromosome segregation and cytokinesis.^2^ Second, RAB24 is primarily involved in basal autophagy and may not be critical under stress-induced autophagic conditions.

We further investigated the mechanistic role of RAB24 in the EMT process. A protein–protein interaction (PPI) network was constructed using genes highly correlated with RAB24, and the core module of this network was identified (Fig. S5A). Within this module, MMP11 has emerged as a key gene of interest due to the well-established role of the matrix metalloproteinase (MMP) family in promoting EMT in cancer. The expression of MMP11 was highly correlated with that of RAB24 in the TCGA-KIRC cohort (Fig. S5B). Interestingly, RAB24 and MMP11 cannot mutually affect each other's expression levels (Fig. S5C). Furthermore, immunofluorescence confirmed that regardless of RAB24 or MMP11 knockdown, their co-localization was obviously visible in the cytoplasm (Fig. S5D).

To investigate their potential interaction, we performed a series of co-immunoprecipitation (Co-IP) assays, which confirmed the presence of both RAB24 and MMP11 proteins in the immunoprecipitated complexes, indicating a possible physical interaction (Fig. S4F–S4I). Functionally, MMP11 overexpression in 786-O cells enhanced cell migration and invasion, effects that were partially reversed by RAB24 knockdown (Fig. S4J). Similar results were observed in Caki-1 cells (Fig. S4K). These findings support a novel EMT-related mechanism involving a synergistic interaction between RAB24 and MMP11.

Autophagy is intricately linked to tumor biology, with the loss or dysfunction of key autophagy regulators increasing cancer susceptibility and progression.^3^ In this study, we provide the first evidence for the oncogenic role of RAB24 in ccRCC, which is driven by two underlying mechanisms. First, RAB24 is essential for cell division, regulating critical mitotic events such as chromosome segregation and cell division.^2^ Second, RAB24 maintains basal autophagy, which confers a survival advantage to cancer cells when they disseminate to secondary organs.^4^

Personalized medicine relies on accurate prognostic assessment, yet the current Tumor Node Metastasis (TNM) staging system lacks sufficient granularity to stratify survival outcomes in patients with locally advanced ccRCC. For example, no significant differences in overall or cancer-specific survival were observed between ccRCC patients with T2bN0M0 and those with T3bN0M0.^5^ This highlights the limitations of TNM staging alone. In this study, we demonstrated that RAB24 expression significantly enhanced the predictive accuracy and clinical utility of TNM staging.

## Ethics approval and informed consent

The animal study was reviewed and approved by the Ethics Committee of the Second Affiliated Hospital of Xi'an Jiaotong University. Consent was obtained from patients for the use of patient data.

## CRediT authorship contribution statement

**Fangshi Xu:** Writing – review & editing, Writing – original draft, Visualization, Software, Methodology, Investigation, Funding acquisition, Formal analysis. **Qiao Du:** Writing – review & editing, Writing – original draft, Visualization, Validation, Methodology, Investigation, Formal analysis. **Gang Li:** Writing – original draft, Visualization, Validation, Software, Resources. **Lingyu Guo:** Writing – review & editing, Writing – original draft, Visualization, Software, Resources. **Jiancang Ma:** Writing – review & editing, Supervision, Project administration, Conceptualization. **Zhenlong Wang:** Writing – review & editing, Supervision, Project administration, Funding acquisition, Conceptualization.

## Data availability

The datasets used and/or analyzed in the current study are available from the corresponding author upon reasonable request.

## Funding

This study was supported by the 10.13039/501100007128Natural Science Foundation of Shaanxi Province, China (No. 2024JC-YBQN-0905) and the 10.13039/501100015401Key Research and Development Program of Shaanxi Province, China (No. 2022SF-464).

## Conflict of interests

The authors declare that they have no competing interests.

## Figures and Tables

**figs1:**
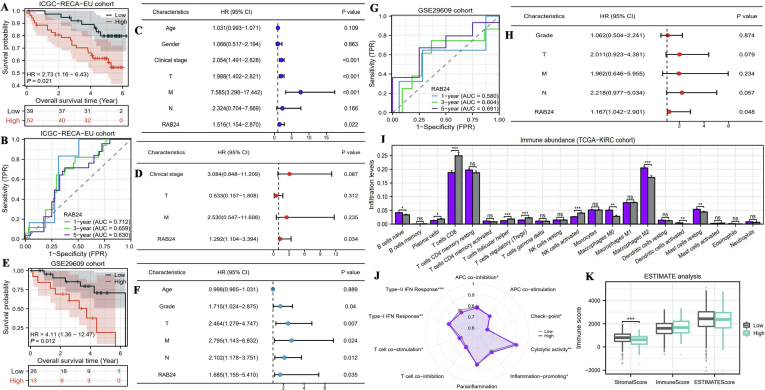


**figs2:**
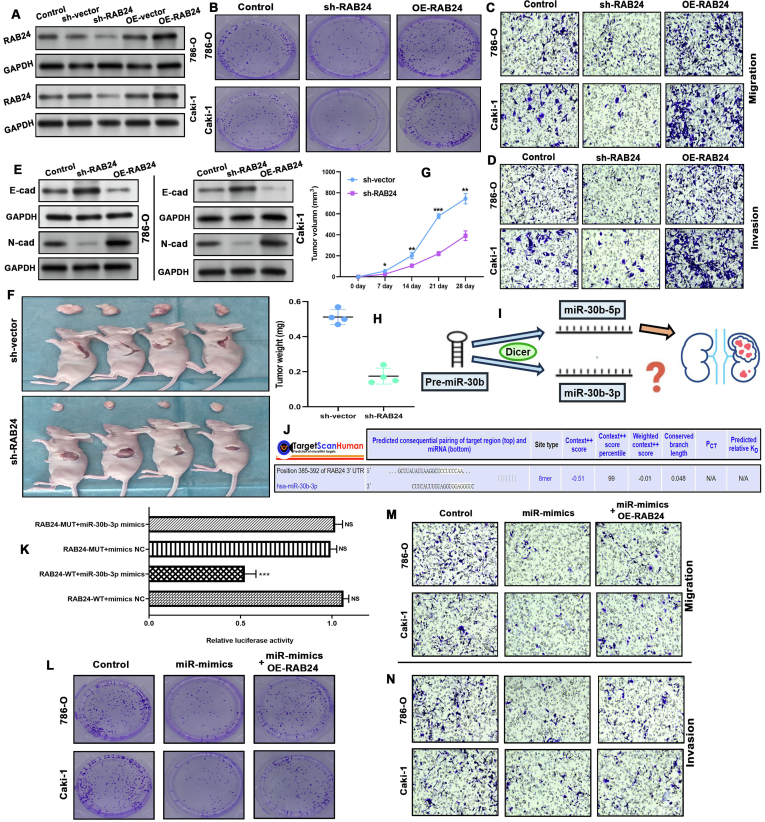


**figs3:**
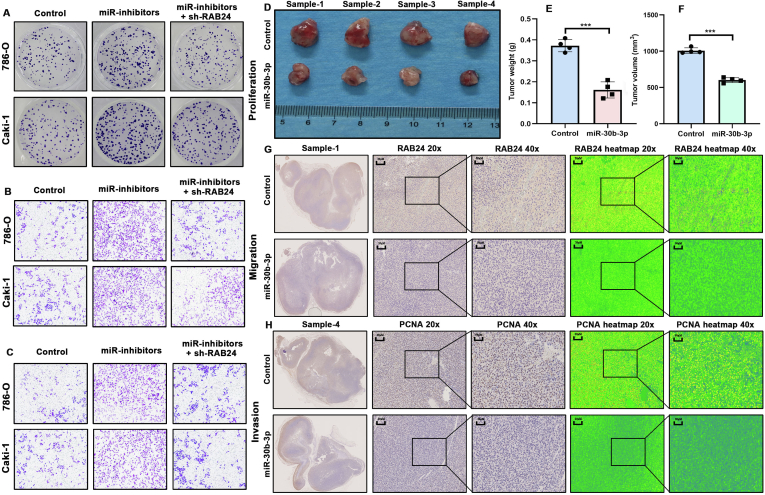


**figs4:**
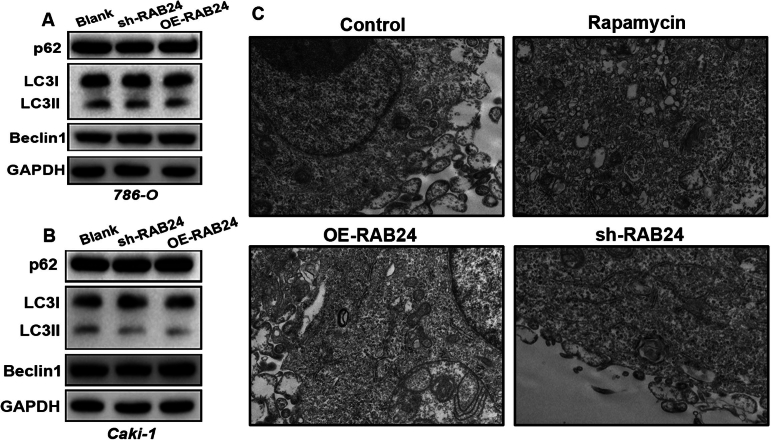


**figs5:**
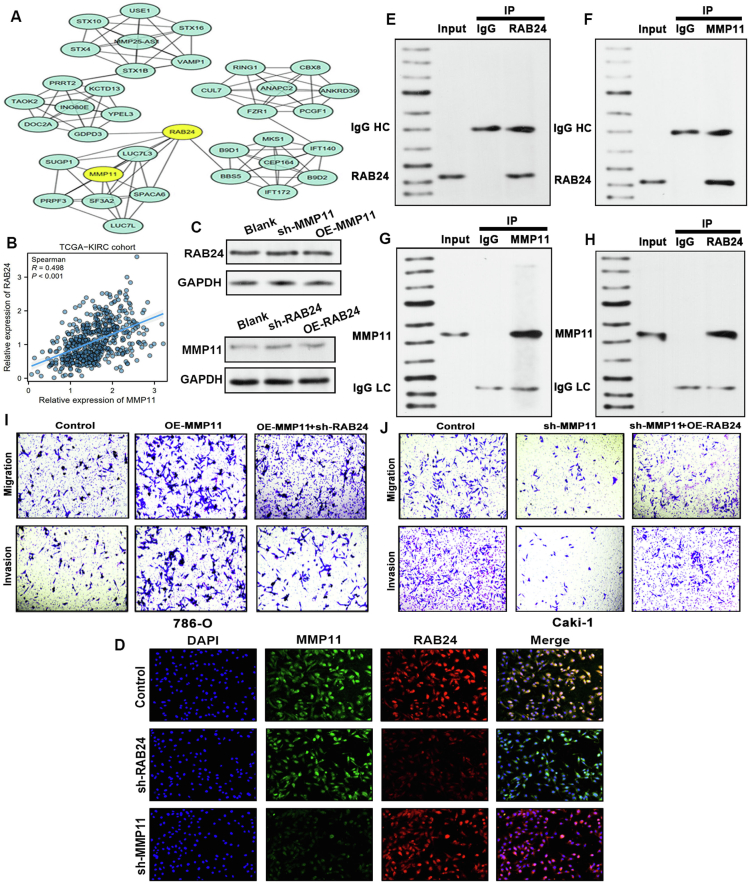


**figs6:**