# Supplementary material for: RAB24 maps comprehensive clinical landscapes and mediates tumor malignant progression under the epigenetic regulation of miR-30b-3p and MMP11 in clear cell renal cell carcinoma
Source: Genes Dis. 2025 Sep 24;13(4):101869. doi: 10.1016/j.gendis.2025.101869 (PMC12999284; doi:10.1016/j.gendis.2025.101869)
Supplement: Multimedia component 1 [file mmc1.docx]

**Supplementary file 1** The specific materials and methods of this study.

**Supplementary figure 1** Validation of the prognostic value of RAB24 in external public cohorts. **(A)** Survival difference between the high- and low- RAB24 expression groups in ICGC-RECA-EU cohort; **(B)** Time-dependent predictive accuracy of RAB24 in ICGC-RECA-EU cohort; **(C, D)** Independent prognostic factors of ccRCC in ICGC-RECA-EU cohort; **(E)** Survival difference between the high- and low- RAB24 expression groups in GSE29609 cohort; **(F)** Time-dependent predictive accuracy of RAB24 in GSE29609 cohort; **(G, H)** Independent prognostic factors of ccRCC in GSE29609 cohort; **(I)** The differential abundances of 22 immune cells between high- and low-RAB24 expression groups; **(J)** The differences in activities of 10 immune signaling pathways between the high- and low- RAB24 expression groups; **(K)** The immune scores of different RAB24 expression groups based on the ESTIMATE method; **P* < 0.05, ***P* < 0.01, and ****P* < 0.001; NS, not significant; ESTIMATE, Estimation of STromal and Immune cells in MAlignant Tumours using Expression data.

**Supplementary figure 2** miR-30b-3p inhibits ccRCC malignant behaviors through targeting RAB24. **(A)** Transfection efficiency of the recombinant vectors of RAB24; **(B)** The effects of RAB24 on ccRCC proliferation via colony formation assays; **(C)** The effects of RAB24 on ccRCC migration via Transwell migration assays; **(D)** The effects of RAB24 on ccRCC invasion via Transwell invasion assays; **(E)** The effects of RAB24 on EMT process via Western blot assays; **(F)** Silencing RAB24 suppresses ccRCC tumor growth in xenograft models; **(G)** Differences in tumor volumes between the sh-vector and sh-RAB24 groups; **(H)** Differences in tumor weight between the sh-vector and sh-RAB24 groups; **(I)** Two miR-30b isoforms tightly regulate ccRCC progression; **(J)** Predicting potential miRNA regulators of RAB24 via the TargetScanHum database; **(K)** The results of dual-luciferase assays; **(L)** miR-30-3p weakens the promotive effects of RAB24 on ccRCC proliferation via colony formation assays; **(M)** Effects of the miR-30-3p/RAB24 axis on ccRCC migration via Transwell migration assays; **(N)** Effects of the miR-30-3p/RAB24 axis on ccRCC invasion via Transwell invasion assays. These experiments were repeated independently three times; Cell quantitative analysis was conducted using a high magnification microscope (100-fold) of five random visual fields; Differences between different groups were assessed using Student’s *t*-test; NC, negative control; MUT, mutation type; WT, wild type; **P* < 0.05, ***P* < 0.01, and ****P* < 0.001.

**Supplementary figure 3** *In vivo* evidence that miR-30b-3p targets RAB24. **(A)** Colony formation rescue experiments of miR-30b-3p inhibitors; **(B, C)** Transwell rescue experiments of miR-30b-3p inhibitors; **(D)** miR-30b-3p mimics inhibits ccRCC tumor growth in xenograft models; **(E, F)** Differences in tumor weight and volume between the control and miR-30b-3p groups; **(G, H)** IHC on xenografts confirmed the differences in RAB24 and PCNA expression between different groups; Xenografts in the control group were established with 786-O cells; Xenografts in the miR-30b-3p group were established with 786-O cells transfected by miR-30b-3p mimics. These experiments were repeated independently three times; Cell quantitative analysis was conducted using a high magnification microscope (100-fold) of five random visual fields; Differences between different groups were assessed using Student’s *t*-test; PCNA, proliferating cell nuclear antigen; Scale bar is 20 or 50 µm in visual fields at different magnifications; ****P* < 0.001.

**Supplementary figure 4** Effects of RAB24 on the autophagy process in RCC. **(A, B)** Effects of RAB24 on the autophagy markers based on Western blot assays; **(C)** Observation of the effect of RAB24 on the autophagy process in 786-O cells using TEM. Rapamycin is used as an autophagy inducer; TEM, transmission electron microscope; The red circle indicates typical autophagosomes.

**Supplementary figure 5** The RAB24-MMP11 interaction drives the EMT process in ccRCC. **(A)** The core module in the PPI network of genes highly correlated with RAB24; **(B)** The expressive correlations between MMP11 and RAB24 (TCGA-KIRC cohort); **(C)** The regulatory relationships between RAB24 and MMP11 based on Western blot assays; **(D)** Co-localization of RAB24 and MMP11 in 786-O cells confirmed by immunofluorescence; **(E–H)** The results of Co-IP assays; **(I)** The effects of the RAB24‬–MMP11 interaction on ccRCC migration and invasion in 786-O cells; **(J)** The effects of the RAB24‬–MMP11 interaction on ccRCC migration and invasion in Caki-1 cells. These experiments were repeated independently three times; Cell quantitative analysis was conducted using a high magnification microscope (100-fold) of five random visual fields; Differences between different groups were assessed using Student’s *t*-test; PPI, protein‬–protein interaction; IP, immunoprecipitation; Co-IP, Co-immunoprecipitation; HC, heavy chain; LC, light chain; ****P* < 0.001.

**Supplementary figure 6** The potential upstream transcriptional regulatory mechanisms of RAB24 in ccRCC progression. **(A)** Two public databases predict TFs regulating RAB24; **(B)** Expressive correlations between RAB24 and candidate TFs (TCGA-KIRC cohort); **(C)** RAB24 is encoded by the negative-sense strand of chromosome 5; **(D)** Binding sites with the top 5 predicted scores based on the JASPAR database; **(E)** The locations of predicted binding sites upstream of the RAB24 TSS; **(F)** The regulatory network of RAB24 in ccRCC progression based on our findings. TFs, transcription factors; TSS, transcription start site; EMT, epithelial–mesenchymal transition.
