# Supplementary material for: RAB24 maps comprehensive clinical landscapes and mediates tumor malignant progression under the epigenetic regulation of miR-30b-3p and MMP11 in clear cell renal cell carcinoma
Source: Genes Dis. 2025 Sep 24;13(4):101869. doi: 10.1016/j.gendis.2025.101869 (PMC12999284; doi:10.1016/j.gendis.2025.101869)
Supplement: Multimedia component 2 [file mmc2.docx]

**Materials and methods**

1. Data source

The clinical information and transcriptomic data of TCGA-KIRC (n=531), ICGC-RECA-EU (n=91) and GSE29609 cohorts (n=39) were utilized for bioinformatics analyses. The transcriptomic data of 28 normal kidney that was obtained from GTEx database merged with that of TCGA-KIRC cohort to screen differentially expressed genes (DEGs). All transcriptome data were standardized using log2 (FPKM+1) transformation.

2. Constructing an autophagy-related gene set

Molecular Signatures Database (MSigDB) (<https://www.gsea-msigdb.org/gsea/msigdb/>) and Human Autophagy Database (HADb) (<http://www.autophagy.lu/>) were applied for the construction of an autophagy-related (AR) gene set.

3. Identification of core autophagy gene in ccRCC

A three-step process was adopted to identify the critical autophagy gene in ccRCC oncogenesis. First, the DEGs between normal and tumor samples were obtained using ‘Limma’ R package under the screening criteria of the absolute value of Log2FC≥1. Next, two machine learning methods assisted us to screen the potential critical autophagy genes in ccRCC, including lasso regression analysis and support vector machine recursive feature elimination (SVM-RFE). Third, the associations of candidate autophagy genes with various cancer-related biological processes, such as angiogenesis, cell cycle, mesenchymal-epithelial transition (EMT) were quantified using CancerSEA database (<http://biocc.hrbmu.edu.cn/CancerSEA/>).

During lasso regression process, the strength of regularization penalization is determined by the parameter lambda. 10-fold cross-validation is used to balance computational efficiency and stability. Mean squared error (MSE) is applied for model evaluation. The maximum number of iterations is 1000. During SVM-RFE process, linear kernel determines the mapping method of data in high-dimensional space and the accuracy of feature ranking. Large step length is used to control the number of features removed in each iteration, and determining the computational efficiency and stability. 10-fold cross-validation is applied to evaluate the generalization ability of the feature subset to avoid overfitting. Features are sorted by the absolute value of their weights and remove the features with the smallest weights.

4. Survival analyses

The ccRCC samples with a short follow-up duration (< 30 days) were excluded. The optimal cutoff value of RAB24 expression was determined using the Cutoff Finder online tool (<http://molpath.charite.de/cutoff>), by which TCGA-KIRC cohort was divided into high-RAB24 and low-RAB24 expression groups. Survival difference analysis was based on the Kaplan-Meier method. The independent prognostic factors of ccRCC were identified using Cox univariate and multivariate analyses. The predictive accuracy of RAB24 was evaluated using the receiver operating characteristic curve (ROC). Decision curve analysis (DCA) was applied to assess if RAB24 could augment the decision benefit of traditional prognostic models. ICGC-RECA-EU (n=91), GSE29609 (n=39) and our center cohort (XJTU cohort, n=20) served as the validation cohorts to test the prognostic value of RAB24.

5. Immune bioinformatic analyses

The associations of RAB24 expression with the infiltration levels of 21 immune cells and the activities of 10 immune-related pathways were assessed using CIBERSORT and ssGSEA algorithms.

6. Clinical samples

The clinical data of 22 ccRCC patients who visited in the department of urology, second affiliated hospital of Xi'an Jiaotong University were collected with their consent (December 2019 to December 2021). No patients were diagnosed with metastatic cases at first visit and all patients received a 2-year follow up. Their tumor node metastasis classification (TNM) and disease status were collected. Tumor progression was defined as local recurrence or distant metastasis confirmed by imaging. This study was approved by the Ethics Committees of the second affiliated hospital of Xi'an Jiaotong University.

7. Cell culture and transfection

Two ccRCC cells (786-O and Caki-1) and a renal tubular epithelial cell (HK-2) were applied in experiments in vitro, which were purchased from Procell company (Wuhan, China). The specific short hairpin RNA (shRNA) for knocking down RAB24 (sh-RAB24) and RAB24 overexpression vectors (OE-RAB24) were designed and synthesized by HanHeng Biotechnology (Shanghai, China).

8. Quantitative real-time polymerase chain reaction (qRT-PCR)

Total RNA was extracted using TRIzol reagent (TaKaRa, Japan). The optical density (OD) at 260 nm and 280 nm of the sample was measured using an ultraviolet spectrophotometer (Nanodrop 2000 spectrophotometer). Reverse transcription was conducted using the PrimeScript RT reagent kit (TaKaRa, Japan). qRT-PCR analysis was performed using SYBR-Green PCR Reagent (Takara, Japan) on the ABI Prism 7900 system. GAPDH was used as the internal reference. The relative gene expression was calculated using the 2^−ΔΔCT^ method.

9. Western blot

The transfected cells were lysed on ice with RIPA buffer (Beyotime, China). BCA kit (Beyotime, China) was used to measure the protein concentration of samples. Proteins were separated by 10% SDS-PAGE (Applygen, China) and electrophoresed on PVDF membranes (Absin, China). The membranes were blocked by 5% skim milk and were washed by TBST buffer (Absin, China). Protein blots were detected using BeyoECL plus solution (Beyotime, China) after incubation with primary and secondary antibodies (Abcam, China). The primary antibodies were as follows: anti-RAB24 rabbit monoclonal antibody (1/1000, ab154824), anti-E Cadherin rabbit monoclonal antibody (1/1000, ab212059), anti-N Cadherin rabbit monoclonal antibody (1/5000, ab76011), anti-MMP11 rabbit polyclonal antibody (1/1000, ab53143), and anti-GAPDH rabbit polyclonal antibody (1/2500, ab9485).

10. Immunohistochemical staining (IHC)

IHC workflow was analogous with our previous study (PMID: 38303549). Briefly, following dehydration, embedding, dewaxing, and rehydration, paraffin sections were treated with 1.5% H2O2 solution in order to inactivate endogenous peroxidase. Antigen repair was accomplished with citric acid buffer. Then, the tissue sections were stained using the ABC method after being blocked by primary and secondary antibodies.

11. Colony formation assay

Cells in log phase growth were seeded in 6-well plates with a density of 1×10^3^ per well. After 2 weeks incubation, the colonies were visible, which were subsequently fixed and stained by Giemsa stain in methanol. Using a microscope, colonies were counted in five random visual fields.

12. Transwell migration and invasion assays

In a 24-well Transwell chamber (Corning, NY, USA), transfected cells were seeded (1 × 10^4^ per well). The medium with 0.1% FBS was added to the upper chambers, while the medium with 10% FBS was added to the lower chambers. After 24h incubation, non-migrated cells were removed using cotton swabs and PBS. Migrated cells were fixed with paraformaldehyde and stained with 0.1% crystal violet. Under a high-magnification microscope, migrated cells were counted in five random visual fields (100-fold). The upper chambers were precoated with Matrigel in Transwell invasion assays.

13. Dual luciferase assay

The recombinant vectors of wild type (WT) and mutation type (MUT) of RAB24 3’UTR region were designed and synthesized by Genechem (Shanghai, China). The experimental flow was similar to the previous description (PMID: 28657147). 293T cells were co-transfected with miRNA and RAB24 vectors. Luciferase reagent (Beyotime, China) was added in lysed cells. The relative fluorescence intensity of sample was measured using a microplate reader.

14. Co-immunoprecipitation (Co-IP)

Protein A+G Agarose (Beyotime, China) was used to perform this experiment, which followed the protocol of manufacturer. Briefly, protein A/G magnetic beads were bound and crosslinked by DSS to the first antibody. Cells were lysed and incubated with beads overnight. Afterward, bound antigens were eluted from the beads by sample buffer and detected using Western blot assay.

15. Xenograft assay

Tumor xenograft experiments were performed on the female BALB/c nude mice aged 6 weeks. Each mouse was subcutaneously injected with 786-O cells transfected with sh-RAB24 or sh-vector. The tumor volume was calculated as follows: tumor volume = 0.5 × (tumor length) × (tumor width)^2^. Once every five days, tumor length and width were measured using a vernier caliper. All mice were euthanized two weeks later for tumor removal. This study was approved by the Ethics Committee of the Second Affiliated Hospital of Xi’an Jiaotong University.

16. Statistical analysis

All statistical analyses were performed using the R software (version 4.1.2) and GraphPad Prism (version 8.0.1). The continuous variables between the groups were compared based on the t-test or Kruskal-Walli’s test. The categorical variables were compared based on the Wilcoxon rank sum test. Correlation analyses were based on the Spearman method. A three-time independent in vitro experiment was conducted. A statistically significant difference was considered to be P＜0.05.
